# Defect-State Engineering in Doped CeO_2_ for Oxygen Storage: Aliovalent Substitution, Co-Doping, and Pathway-Dependent Regulation

**DOI:** 10.3390/molecules31111896

**Published:** 2026-06-01

**Authors:** Yaohui Xu, Quanhui Hou, Yunxuan Zhou, Zhao Ding

**Affiliations:** 1 Laboratory for Functional Materials, School of New Energy Materials and Chemistry, Leshan Normal University, Leshan 614000, China; xyh1986@lsnu.edu.cn; 2 Leshan West Silicon Materials Photovoltaic and New Energy Industry Technology Research Institute, Leshan 614000, China; 3 School of Automotive Engineering, Yancheng Institute of Technology, Yancheng 224051, China; 4 Lanxi Magnesium Materials Research Institute, Lanxi 321100, China; yunxuanzhou@cqu.edu.cn; 5 National Engineering Research Center for Magnesium Alloys, National Innovation Centre for Industry-Education Integration of Energy Storage Technology, College of Materials Science and Engineering, Chongqing University, Chongqing 400044, China

**Keywords:** CeO_2_, oxygen storage capacity, defect engineering, oxygen vacancies, rare-earth doping, cation–anion co-doping

## Abstract

CeO_2_ is a representative oxygen-storage oxide because its fluorite lattice can reversibly release and reincorporate oxygen through the Ce^4+^/Ce^3+^ redox couple and the associated formation and annihilation of oxygen vacancies. Although doped CeO_2_ has been studied extensively, the literature has often treated oxygen-storage enhancement mainly in terms of dopant identity and composition, whereas the more fundamental issue is how a given doping strategy constructs a specific defect state within the fluorite host. Here, oxygen-storage enhancement is discussed from the standpoint of defect-state engineering. The discussion focuses on three routes, as follows: rare-earth single doping, cation–anion co-doping, and route-dependent dopant incorporation. Rare-earth single doping correlates aliovalent substitution with lattice expansion, vacancy generation, and finite oxygen-storage-capacity (OSC) optima. Cation–anion co-doping further shows that simultaneous perturbation of the cationic and anionic sublattices can amplify the defect response, while also demonstrating that vacancy concentration alone does not fully account for OSC enhancement. Route-dependent doping adds an additional dimension by showing that the same dopant can produce different lattice responses, defect populations, and oxygen-release behaviors when introduced through different pathways. On this basis, the review argues that OSC in doped CeO_2_ is more meaningfully rationalized through a coupled descriptor set involving lattice accommodation, Ce^3+^/Ce^4+^ redistribution, oxygen-vacancy abundance, and dopant incorporation pathway. Taken together, these observations shift the design logic of oxygen-storage ceria from empirical dopant screening toward deliberate defect-state construction.

## 1. Introduction

Cerium dioxide (CeO_2_) is a representative oxygen-storage oxide because its fluorite lattice can reversibly release and reincorporate oxygen through the Ce^4+^/Ce^3+^ redox couple and the associated formation and annihilation of oxygen vacancies [1,2,3]. This redox-buffering capability underpins the widespread use of ceria-based materials in three-way catalysis and related oxygen-transfer processes, and also makes CeO_2_ a valuable model system for understanding how defect chemistry governs function in reducible oxides [4,5]. From a materials-design perspective, the importance of CeO_2_ does not lie simply in its thermal or chemical stability, but in the fact that lattice oxygen can participate reversibly in redox processes without destroying the basic fluorite framework. Oxygen storage capacity (OSC), therefore, should be viewed not merely as an empirical performance index, but as a functional manifestation of the defect-responsive nature of ceria. For this reason, CeO_2_ remains a particularly instructive oxide for a focused discussion. Beyond oxygen-buffering catalysis, ceria-based materials are relevant to gas sensing, solar-thermochemical redox chemistry, oxygen-transport-related electrochemical systems, and other applications in which the accessibility, reversibility, and mobility of lattice oxygen govern function. In many of these settings, the practical challenge is not simply to increase vacancy concentration, but to control how oxygen-active defect states are formed, coupled to cation redox response, and maintained under operating conditions.

The oxygen-storage behavior of CeO_2_ originates from the non-stoichiometric response of the fluorite lattice under reducing and oxidizing atmospheres. Under oxygen-deficient conditions, partial reduction in Ce^4+^ to Ce^3+^ is accompanied by the release of lattice oxygen and the generation of oxygen vacancies, yielding non-stoichiometric CeO_2−x_; upon reoxidation, these vacancies can be replenished and the cerium cations restored toward the Ce^4+^ state [6,7]. Because this process proceeds without a reconstructive phase transformation, ceria can sustain repeated oxygen release/storage cycles while largely retaining its crystallographic framework. The central issue in CeO_2_ oxygen storage is thus not oxygen adsorption in a simple surface sense, but the coupled evolution of vacancy concentration, redox-active cerium species, and lattice accommodation during reversible oxygen exchange [8]. This intrinsic coupling is what gives ceria its technological importance in oxygen-buffering applications and also what makes doped CeO_2_ a particularly suitable system for mechanistic analysis.

Once aliovalent dopants are introduced into CeO_2_, this intrinsic defect equilibrium is deliberately perturbed. Substitution of Ce^4+^ by lower-valence cations creates charge-compensating extrinsic oxygen vacancies and modifies the local Ce^3+^/Ce^4+^ distribution, while anion doping can further disturb the oxygen sublattice and reshape the surrounding electronic structure [9]. Doping has therefore long been regarded as one of the most effective routes for improving the oxygen-storage properties of ceria-based materials. However, the literature has often discussed this problem in composition-centered terms, that is, in terms of which dopant is used and how much is added. Such a description is useful at an exploratory stage, but it does not fully explain why some doped CeO_2_ systems exhibit clear OSC optima, why co-doping can outperform single doping, or why the same dopant can behave differently when introduced through different synthesis pathways. The more fundamental issue is how a given doping strategy constructs a particular defect state within the fluorite host [10,11]. In defect-chemical terms, lower-valence acceptor doping may be represented in Kröger–Vink notation as RE_2_O_3_ → 2RE_Ce_^′^ + V_O_^••^ + 3O_O_^x^, illustrating how aliovalent substitution promotes oxygen-vacancy formation in the fluorite host. By contrast, higher-valence donor-type substitution can shift the compensation balance in a different direction, for example by favoring electronic compensation or suppressing vacancy formation, depending on composition and oxygen chemical potential. In this sense, doping in ceria is not limited to vacancy enrichment by lower-valence cations, but can modulate reducibility, transport, catalytic response, and related functional properties through multiple compensation pathways. Although the present perspective focuses on acceptor-type single doping, cation–anion co-doping, and pathway-dependent incorporation, this broader defect-chemical landscape should be kept in mind when interpreting how aliovalent perturbation modifies oxygen-storage behavior [10,11,12].

This distinction matters because CeO_2_ has already been reviewed extensively from broad perspectives, including synthesis, morphology control, catalysis, environmental remediation, and general energy-related applications. Another panoramic overview would therefore add limited value. What remains less sharply defined is a narrower, mechanism-oriented discussion in which oxygen-storage enhancement is interpreted explicitly as a problem of defect-state engineering. Recent studies have now provided more systematic datasets linking aliovalent doping to lattice response, oxygen-vacancy generation, Ce^3+^/Ce^4+^ redistribution, and quantified OSC [13,14,15]. More importantly, these studies indicate that OSC in doped CeO_2_ cannot be rationalized satisfactorily by any single parameter alone, whether dopant content, vacancy concentration, or specific surface area. Instead, oxygen-storage behavior appears to emerge from a coupled defect-state network shaped jointly by lattice accommodation, redox flexibility, oxygen-vacancy abundance, and dopant incorporation history.

**Figure 1:**
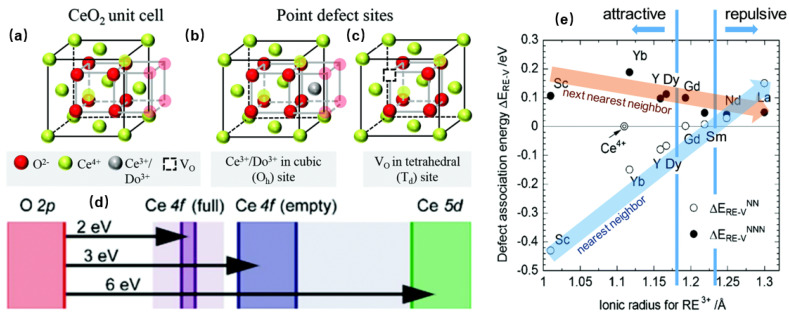
Defect chemistry of ceria relevant to oxygen storage. (**a**) Fluorite CeO_2_ unit cell showing the basic arrangement of Ce and O sublattices. (**b**) Representative Ce^3+^-containing point-defect configuration in the fluorite lattice, illustrating local charge compensation and coordination distortion after reduction or aliovalent perturbation. (**c**) Representative oxygen-vacancy configuration, showing how vacancy formation changes the local coordination environment and creates an oxygen-accessible defect site. (**d**) Idealized O 2p–Ce 4f–Ce 5d energy-level alignment, highlighting the electronic basis for Ce^4+^/Ce^3+^ redox redistribution during oxygen release and reincorporation. (**e**) Dependence of rare-earth dopant–vacancy association energy on dopant ionic radius, illustrating that vacancy accessibility is affected by dopant size and dopant–vacancy binding tendency. Overall, the figure emphasizes that oxygen storage in doped CeO_2_ is governed by coupled redox redistribution, vacancy formation/accessibility, defect association, and lattice accommodation rather than by vacancy concentration alone. Combined from Ref. [16].

Two points frame the discussion. First, as summarized in Figure 1, oxygen storage in ceria is rooted in coupled defect chemistry rather than in vacancy count alone. Dopant-vacancy association, Ce^3+^/Ce^4+^ redistribution, local coordination geometry, and lattice accommodation jointly determine whether a nominally introduced defect becomes thermodynamically accessible and functionally reversible. Second, the three case studies emphasized below are treated as representative platforms rather than as the sole evidentiary basis of the field. Rare-earth single doping serves here as a baseline case for aliovalent substitution, cation–anion co-doping is used to examine whether simultaneous perturbation of both sublattices amplifies the coupled defect response, and route-dependent yttrium incorporation is used to show that nominal dopant identity does not uniquely determine the final defect state [13,14,15]. The aim is therefore not to restate these studies one by one, but to extract an interpretive framework in which lattice accommodation, redox redistribution, oxygen-vacancy abundance, and incorporation pathway are considered together.

The discussion therefore focuses on oxygen storage in doped CeO_2_ from the standpoint of defect-state engineering, rather than attempting to catalogue all doped ceria systems or all applications of CeO_2_. The discussion begins with rare-earth single doping as a baseline case for identifying how aliovalent substitution reorganizes the fluorite lattice and produces finite OSC optima. It then turns to cation–anion co-doping, where the oxygen-storage response becomes more explicitly coupled to both lattice distortion and defect amplification. The third case study examines route-dependent dopant incorporation to show that nominal dopant identity does not uniquely determine the final defect state. On this basis, the review finally considers which descriptors are genuinely informative for rationalizing OSC in doped CeO_2_ and argues that a coupled descriptor set, rather than any single structural parameter, provides the more meaningful framework for future design. In this way, oxygen-storage research on doped CeO_2_ is placed on a clearer mechanistic footing, with dopants treated as tools for constructing defect states and synthesis routes treated as part of the design space rather than as background procedure. The discussion here remains focused on CeO_2_. Although other oxygen-storage oxides, including perovskite-type systems, are also scientifically important, ceria remains the clearest fluorite model system in which lattice accommodation, redox redistribution, oxygen-vacancy accessibility, and incorporation history can be discussed within a comparatively coherent defect-chemical framework. Broader cross-family comparison is valuable, but it lies beyond the scope of this discussion.

## 2. Rare-Earth Single Doping: Ionic Radius, Lattice Expansion, and Finite Optima

Rare-earth single doping offers a useful starting point for understanding how aliovalent substitution modifies oxygen storage in CeO_2_. When trivalent rare-earth cations replace Ce^4+^ in the fluorite lattice, charge imbalance and local size mismatch promote the formation of extrinsic oxygen vacancies, alter the Ce^3+^/Ce^4+^ distribution, and induce measurable lattice expansion [17,18,19]. At the same time, this response is not indefinite. Rather than increasing monotonically with dopant content, the oxygen storage capacity (OSC) of doped CeO_2_ commonly reaches a finite optimum, indicating that the relevant structural regime is governed by the coupled evolution of solid solubility, local distortion, and defect-state density [20,21]. Here, “finite optimum” refers to the dopant concentration within a given synthesis series at which the measured OSC reaches a maximum before declining again at higher loading. In this sense, rare-earth single doping is useful not merely as an elemental comparison, but for identifying which structural and defect descriptors are genuinely relevant to OSC enhancement.

**Figure 2:**
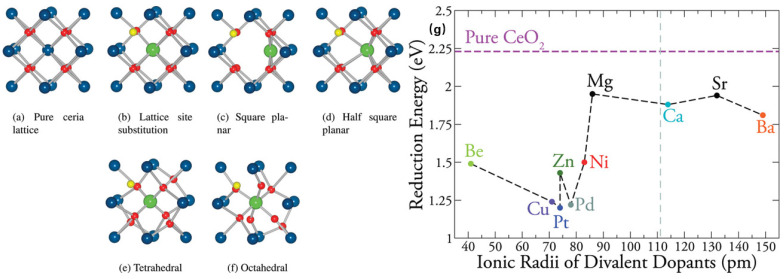
Lattice accommodation in doped ceria. (**a**–**f**) Representative relaxed defect motifs in doped CeO_2_, illustrating how aliovalent substitution and oxygen-vacancy formation generate dopant-specific local coordination environments within the fluorite lattice. Panel (**a**) shows a reference local configuration for dopant incorporation; panels (**b**–**f**) compare representative dopant–vacancy arrangements with different degrees of local relaxation, coordination distortion, and lattice accommodation. (**g**) Dependence of ceria reduction energy on dopant ionic radius, showing that reducibility is not governed by dopant valence alone but varies nonmonotonically with dopant size and lattice accommodation. Overall, the figure emphasizes that oxygen storage in doped CeO_2_ should be interpreted through the coupled effects of vacancy formation, dopant–vacancy association, local lattice distortion, and reduction energetics. Combined from Ref. [10].

Comparative rare-earth-doped CeO_2_ series prepared under closely matched conditions provide a particularly useful basis for this discussion because dopant identity and concentration can be varied without simultaneously changing the broader host architecture. In representative porous fluorite systems, Yb, Y, Sm, and La are introduced into CeO_2_ through the same solvothermal–calcination sequence, while the multilayered mesoporous character of the parent CeO_2_ is largely retained [13]. This matters because it allows the oxygen-storage response to be interpreted mainly in terms of lattice incorporation and defect-state evolution rather than gross morphology reconstruction. More broadly, such composition-controlled series are especially informative because they separate the effect of aliovalent substitution from changes in synthesis background, an issue that often complicates cross-comparison among different doped-ceria studies [17,20]. This broader dependence on accommodation is not unique to the rare-earth series discussed here. Across doped-ceria studies more generally, OSC enhancement remains bounded by how effectively the fluorite host absorbs local strain, stabilizes substitutional incorporation, and couples vacancy formation to a redox-accessible cerium sublattice. The rare-earth dataset therefore serves as a baseline case for the broader defect-chemistry pattern discussed here rather than as an isolated materials list. The structural logic behind this baseline case is summarized in Figure 2, which highlights representative lattice-accommodation modes and the nonmonotonic relation between dopant size and reducibility in doped ceria.

The first level of response to rare-earth incorporation is structural. In the source study, these patterns were assigned to fluorite-type CeO_2_ by comparison with the standard CeO_2_ pattern (JCPDS no. 34–0349), and the reported phase assignment is adopted here, without introducing a separate diffraction refinement. The emphasis therefore remains on the structural trend and its mechanistic implication rather than on software-specific indexing workflows or an artificial unified refinement table assembled from independent source studies. At the same time, the fluorite reflections shift systematically toward lower diffraction angles after rare-earth incorporation, showing that lattice expansion is real as well as composition-dependent rather than a merely nominal consequence of dopant addition. This matters because the structural consequence of doping, not only dopant identity, conditions the defect state that later contributes to oxygen release and reincorporation. The different beneficial windows observed for Yb, Y, Sm, and La should therefore not be attributed to formal valence, which is the same for all four dopants, but to how each ion couples its size mismatch and vacancy-binding tendency to the fluorite host. Ionic radius, elastic strain, local coordination preference, and the strength of dopant-vacancy association all influence how far substitution can proceed before coherent accommodation is lost. In this sense, rare-earth solubility in CeO_2_ is not set by charge alone, but by the total energetic cost of incorporating a given trivalent ion into a defect-bearing fluorite lattice. This also explains why lattice expansion and oxygen-vacancy formation are not contradictory trends. Removing an O^2−^ ion locally relaxes coordination, but the dominant average crystallographic response can still be expansion because replacement of Ce^4+^ by the larger RE^3+^ ion and the accompanying relaxation of neighboring Ce–O polyhedra outweigh the volume contraction associated with a single vacancy.

Local-structure observations reinforce the same interpretation. High-resolution structural analysis indicates that rare-earth substitution increases the interplanar spacing of CeO_2_, again consistent with lattice expansion after incorporation of larger trivalent ions. At the same time, the porous framework of the parent CeO_2_ remains largely preserved after rare-earth introduction. These observations indicate that the subsequent performance changes cannot be reduced to a transition into a completely different host structure. A more reasonable interpretation is that rare-earth incorporation reorganizes the local crystal chemistry and defect population within a structurally comparable fluorite framework. In other words, the problem is best framed not as one of morphology replacement, but as one of defect-state construction within an accommodated host lattice [13,18,22,23]. This does not mean that morphology or exposed crystalline faces are irrelevant. Rather, within the composition-controlled rare-earth series emphasized here, morphology remains comparatively preserved within the reported range, and the dominant variable is defect-state reorganization [5,6,7]. The second, and more consequential, level of response is the evolution of defect states. Rare-earth incorporation affects both the cerium redox state and the oxygen-vacancy population in a coordinated way [13]. The Ce^3+^ fraction rises from the undoped value to a maximum at intermediate doping, while the vacancy-related Raman response follows the same nonmonotonic trend, indicating that defect enrichment is real but compositionally bounded. This distinction separates beneficial defect construction from defect overload. At low to moderate dopant contents, aliovalent substitution enlarges the redox-active pool and facilitates reversible oxygen exchange; beyond the optimum, continued lattice perturbation no longer translates into proportionally more useful oxygen-storage sites [12]. The key implication is that OSC maxima arise from coupled structural and redox balance, not from monotonic vacancy creation. Beyond the optimum, part of the added dopant may cease to generate reversibly accessible vacancies and instead contribute to stronger defect association, local over-distortion, or incomplete lattice incorporation.

**Figure 3:**
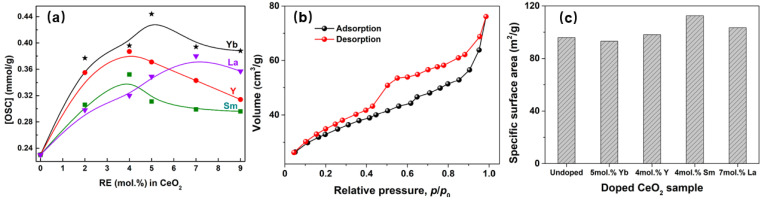
Representative summary of the finite oxygen-storage optima observed in rare-earth-doped CeO_2_. Panel (**a**) compares the OSC trends of representative Yb-, Y-, Sm-, and La-doped series, highlighting the appearance of composition-dependent maxima rather than monotonic performance gains. Panel (**b**) illustrates the preserved adsorption/desorption behavior characteristic of the mesoporous fluorite host. Panel (**c**) compares the specific surface areas of undoped and representative doped samples, showing that the strongest OSC response cannot be explained by BET surface area alone. Combined from Ref. [13].

These structural and defect changes translate directly into OSC enhancement, but the enhancement is not monotonic. As shown in Figure 3, each rare-earth dopant displays a finite optimum rather than an indefinite increase in performance with increasing dopant content [13]. Maximum OSC values are reached only within specific composition windows, which shifts the discussion away from the oversimplified idea that more dopant necessarily means more vacancies and therefore higher OSC. Instead, the available data indicate that oxygen-storage enhancement depends on achieving a favorable balance among lattice accommodation, defect generation, and redox accessibility [20,21]. Figure 3 also shows that the highest OSC cannot be explained by specific surface area alone. Although several doped samples exhibit relatively large BET surface areas, the strongest OSC response does not simply coincide with the largest surface area [13]. This observation indicates that oxygen-storage improvement in RE-doped CeO_2_ is fundamentally a defect-state problem rather than a purely textural one. A similar decoupling between specific surface area and functional response has also been reported in La-modified CeO_2_, where the La-doped material displayed distinct electrical and optical response relative to undoped ceria despite a lower surface area, again illustrating that defect-state reorganization can be more functionally decisive than textural metrics alone [24]. More broadly, it supports the idea that lattice accommodation and redox-state reorganization are more informative than surface area alone when interpreting oxygen-storage behavior in aliovalently substituted ceria [8,17,18].

Taken together, the rare-earth single-doping case already reveals the central logic that recurs throughout the following discussion. First, aliovalent substitution must be evaluated in terms of actual lattice incorporation rather than nominal addition. Second, lattice expansion is not merely a passive crystallographic consequence, but part of the oxygen-storage response because it reflects how the fluorite host accommodates dopant-induced defect formation [18,19,20,21,22]. Third, oxygen-vacancy concentration is essential, but it becomes meaningful only when interpreted together with Ce^3+^/Ce^4+^ redox flexibility and solid-solution behavior. Finally, the existence of finite optima shows that OSC enhancement emerges from a coupled defect-state configuration rather than from a one-directional increase in any single parameter. Rare-earth single doping therefore serves as the baseline from which the more complex cases of co-doping and route-dependent incorporation can be understood.

## 3. Cation–Anion Co-Doping: Synergistic Amplification of Defect States

While rare-earth single doping establishes the basic role of aliovalent substitution, cation–anion co-doping introduces a more strongly coupled mode of defect regulation. In such systems, the modification of CeO_2_ is no longer governed only by cation replacement, but by the simultaneous perturbation of both the cationic and anionic sublattices [25,26]. Once both sublattices are involved, lattice distortion, oxygen-vacancy generation, and the fraction of cerium sites that can reversibly shuttle between Ce^4+^ and Ce^3+^ during reduction-oxidation cycling become more tightly intertwined, making the oxygen-storage response of CeO_2_ a more explicitly coupled defect-engineering problem. In this sense, cation–anion co-doping is important not because it simply adds more dopants, but because it changes the mode by which defect states are constructed within the fluorite host [27,28,29,30].

Composition-controlled Yb/N-co-doped CeO_2_ series make it possible to examine the problem under stepwise and structurally comparable cationic and anionic perturbations. In representative systems of this kind, the Yb content is fixed at 5 mol.% while the nominal nitrogen input, introduced through triethanolamine, is varied over a broader range under otherwise matched solvothermal–calcination conditions [14]. This design separates two issues that are often conflated in the doping literature, namely the following: whether a cation dopant can be incorporated into the fluorite lattice in a structurally coherent way, and whether further perturbation of the anion sublattice can intensify the defect response without immediately destabilizing the host structure. The resulting comparison therefore moves beyond single-substitution chemistry and offers a more explicit test of coupled defect-state construction [27,28,29,30]. More importantly, the Yb/N system is discussed here to address a broader mechanistic question, as follows: once both the cationic and anionic sublattices are deliberately perturbed, which variables still track oxygen-storage behavior most meaningfully, and which merely change in parallel? Framed in this way, the value of the co-doping case lies less in the specific Yb/N combination itself than in its ability to expose how lattice accommodation, redox redistribution, vacancy formation, and structural accommodation become more tightly coupled than in the single-doping case. Other co-doped ceria systems have indeed been reported, but they often vary multiple factors simultaneously, including dopant identity, synthesis route, and loading level, which makes direct mechanistic comparison difficult. The Yb/N series is therefore used here as a deliberately restricted comparison, while the relative scarcity of similarly systematic ceria-based co-doping datasets remains an important gap in the literature. This limitation also defines the boundary of the present discussion, as follows: the co-doping section is intended to clarify a mechanistic pattern, not to imply that the Yb/N system alone is sufficient to generalize all cation-anion co-doping behavior in ceria.

The first level of response to co-doping is structural. Both Yb-doped and Yb/N-co-doped samples retain the cubic fluorite phase, with no detectable impurity phases such as CeN, YbN, or Yb_2_O_3_ [14]. At the same time, the lattice parameters of Yb-doped and Yb/N-co-doped CeO_2_ are larger than those of undoped CeO_2_, indicating that local lattice distortion accompanies the partial substitution of Ce^4+^ and O^2−^ by the larger Yb^3+^ and N^3−^ ions. More importantly, the lattice-parameter trends are not random. In the Yb-only series, the lattice parameter increases up to 5 mol.% Yb, indicating that this composition corresponds to the Yb solid-solubility limit in CeO_2_. Upon subsequent nitrogen introduction, the lattice parameter of 5%Yb-doped CeO_2_ continues to increase almost linearly up to 20%N, suggesting that nitrogen can be incorporated effectively into the Yb-containing fluorite lattice within this range; beyond this point, the lattice parameter decreases again, which is interpreted here as part of a broader oversaturation regime rather than as continued beneficial incorporation [14]. This interpretation is based not on lattice parameter alone, but on the combined structural, textural, defect-sensitive, and performance trends observed at higher nominal N input, including the onset of morphology disruption, the decline in BET surface area, and the loss of continued OSC enhancement. Thus, cation–anion co-doping, like rare-earth single doping, also operates within a finite beneficial window rather than an unlimited compositional range. The key point is not merely that more nitrogen can be introduced, but that only moderate anion perturbation remains compatible with structurally accommodated defect formation; once that balance is exceeded, further nominal input no longer translates into a more useful oxygen-storage state [10,11].

The morphology and porosity results sharpen the same conclusion. The multilayered porous architecture of the parent CeO_2_ remains broadly recognizable after Yb addition and even after moderate N incorporation, but excessive nitrogen input disrupts that structural regime and leads to spheroidal aggregates. The BET results follow the same tendency, as follows: the specific surface area remains comparable between undoped CeO_2_ and 5%Yb-doped CeO_2_, increases only modestly in the beneficial co-doping range, and then drops sharply once the nitrogen level becomes excessive [14]. These observations show that moderate anion introduction can intensify defect-state regulation while preserving the porous fluorite host, whereas excessive perturbation begins to destabilize the structural framework in which those defects are useful. 

Defect-sensitive characterization leads to the same conclusion. XPS confirms the presence of both Yb and N in the co-doped system, and the calculated [Ce^3+^] and [VO] values increase in sequence from undoped CeO_2_ to 5%Yb-doped CeO_2_ and then increase further in the optimally co-doped regime [14]. The Yb contribution can be interpreted in first approximation as acceptor-type substitution that favors oxygen-vacancy formation, but the additional role of nominal nitrogen input should not be presented as a single, unique compensation pathway. In the present dataset, the optimally co-doped samples exhibit stronger Ce^3+^-related XPS intensity and higher vacancy-sensitive Raman response than the Yb-only sample. We therefore interpret N addition pragmatically as part of a co-doping regime that yields a more reduced and defect-richer final state within a limited composition window, rather than as direct proof that substitutional N^3−^ necessarily drives additional Ce^4+^ to Ce^3+^ conversion by itself. Once the nominal N content becomes excessive, incomplete incorporation, defect association, local electronic rearrangement, or incipient segregation can all compete with useful vacancy construction, which is consistent with the subsequent decline in OSC. Raman spectroscopy supports the same conclusion. For ideal fluorite CeO_2_ (space group Fm$\bar{3}$m), first-order Raman scattering is dominated by the F2g mode, typically observed near 460–465 cm^−1^. Additional broad bands in the ~550–600 cm^−1^ region are commonly assigned to defect- or disorder-induced scattering associated with oxygen vacancies, local symmetry breaking, and nonstoichiometric CeO_2−x_ environments. In the present datasets, retention of the F2g band confirms preservation of the fluorite host, whereas the strengthening and modest displacement/broadening of the defect-related band relative to values commonly reported in the literature are consistent with dopant-induced lattice distortion and vacancy enrichment rather than with reconstructive phase transformation. For undoped CeO_2_, the spectrum is dominated by the fluorite F2g mode near 462 cm^−1^, accompanied by a weaker defect-related band near 592 cm^−1^, indicating the presence of intrinsic vacancy defects. After Yb introduction, the defect band becomes much more pronounced, and under optimal Yb/N co-doping it even exceeds the F2g mode in intensity. When the relative vacancy concentration is estimated through the I592/I462 ratio, the value rises almost linearly with increasing nitrogen content in the 5%Yb-doped series and reaches a maximum at the optimal co-doping composition [14]. Here, I592/I462 denotes the ratio between the intensity of the vacancy-related Raman band near 592 cm^−1^ and that of the fluorite F2g band near 462 cm^−1^, and is therefore used as a semi-quantitative measure of the relative defect-state intensity within the CeO_2_ host. In other words, Yb/N co-doping does not merely superimpose two independent substitutions; it intensifies vacancy formation through simultaneous perturbation of both sublattices [27,28,30].

**Figure 4:**
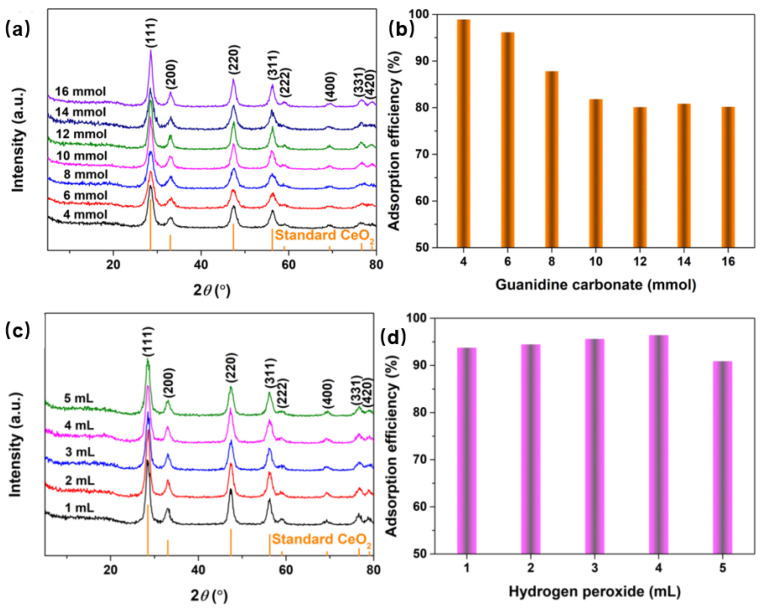
Hydrothermal synthesis parameter effects on CeO_2_ formation and AO7 adsorption performance. (**a**) XRD patterns of CeO_2_ samples hydrothermally synthesized at 180 °C for 24 h with different additions of guanidine carbonate (4–16 mmol) and 5 mL of 30% H_2_O_2_. (**b**) Adsorption efficiencies of AO7 dye for the corresponding CeO_2_ samples shown in panel (**a**) ([CeO_2_] = 2.0 g/L; [AO7] = 100 mg/L; V = 100 mL; 200 rpm; room temperature; no pH preadjustment; t = 60 min). (**c**) XRD patterns of CeO_2_ samples hydrothermally synthesized at 200 °C for 24 h with 4 mmol guanidine carbonate and different additions of 30% H_2_O_2_ (1–5 mL). (**d**) Adsorption efficiencies of AO7 dye for the corresponding CeO_2_ samples shown in panel (**c**) ([CeO_2_] = 2.0 g/L; [AO7] = 110 mg/L; V = 100 mL; 200 rpm; room temperature; no pH preadjustment; t = 60 min). Combined from Ref. [16].

These structural and spectroscopic changes are reflected directly in the reduction behavior and the quantified oxygen-storage response. For undoped CeO_2_, reduction begins around 200 °C and displays two main hydrogen-consumption maxima at approximately 505 and 776 °C, corresponding broadly to surface/subsurface oxygen and bulk oxygen reduction [14]. After Yb doping and especially after Yb/N co-doping, oxygen release begins below 200 °C, a visible shoulder appears around 400 °C, and the reduction band near 600 °C stands well above the baseline. Quantitatively, the OSC of undoped CeO_2_ is 0.115 mmol O_2_ g^−1^, increases to 0.222 mmol O_2_ g^−1^ after 5 mol.% Yb addition, and reaches a maximum of 0.274 mmol O_2_ g^−1^ in the optimally co-doped state [14]. Beyond that point, the OSC decreases again, consistent with the structural and morphological evidence that the beneficial co-doping regime has already been exceeded. The discussion above relies primarily on the reported XPS, Raman, H2-TPR, and OSC results for the Yb/N-co-doped system [9]. Figure 4 instead illustrates, in a more general way, how changes in precursor chemistry can strongly affect fluorite CeO_2_ formation and the resulting functional behavior in porous CeO_2_ systems [14].

More importantly, this co-doping case shows that the relationship between vacancy content and OSC cannot be reduced to the simple statement that more vacancies lead to higher OSC. When OSC is normalized with respect to specific surface area and correlated with both lattice parameter and oxygen-vacancy concentration, both relations are approximately linear, but the correlation with lattice parameter is stronger than that with [VO] alone. This normalization is used only to suppress first-order textural differences, so that the remaining trend can be compared more directly with lattice accommodation and defect-state descriptors. This changes how OSC enhancement in doped ceria should be discussed. If OSC/SBET tracks lattice parameter more strongly than vacancy concentration alone in the Yb/N system, then the most useful descriptor may not be vacancy content considered in isolation, but a broader structural variable that implicitly contains multiple aspects of defect-state evolution. Lattice expansion in doped CeO_2_ is not simply a geometric consequence of inserting larger aliovalent ions. It also reflects how the fluorite framework accommodates charge compensation, how the local environment of cerium changes, and how oxygen mobility may be modified as the crystal moves away from the stoichiometric host state [10,11,14,30].

Taken together, the co-doping case extends the argument beyond rare-earth single substitution. Single doping already shows that aliovalent substitution can create finite OSC optima through lattice incorporation and defect enrichment. Cation–anion co-doping goes further by showing that defect states can be amplified through simultaneous regulation of both sublattices, but also that this amplification remains bounded by structural accommodation and morphology retention. For this reason, the oxygen-storage response of co-doped CeO_2_ is better understood not as a simple function of vacancy concentration, but as the outcome of a coupled defect-state network involving lattice distortion, Ce^3+^/Ce^4+^ redistribution, oxygen-vacancy abundance, and host-structure stability [8,10,11,14]. This leads directly to the next question, as follows: if defect states depend not only on dopant identity and dopant combination, could they also depend on the pathway by which the dopant is introduced into CeO_2_?

## 4. Route-Dependent Doping: The Same Dopant Does Not Produce the Same Defect State

Dopant identity alone does not determine the final defect structure of CeO_2_. Here, the route-controlled Y-doped series is discussed as a comparison of incorporation-path effects rather than as an isolated yttrium case study. Even when the dopant species is fixed, different incorporation routes can generate distinct local environments, defect populations, and oxygen-release behaviors. This point is often understated in discussions of doped ceria, where nominal composition is usually treated as the main variable, yet it becomes important once the discussion moves from composition-oriented screening to process-sensitive defect engineering. In route-controlled Y-doped CeO_2_ systems, the same yttrium precursor is introduced through different incorporation pathways, including hydrothermal doping, impregnation doping, and combined hydrothermal/impregnation treatment. Such comparisons separate dopant identity from dopant incorporation history and make clear that a nominally identical composition does not necessarily correspond to a single, unique defect state. These three routes do not exhaust the synthesis space of ceria-based systems. Rather, they are selected because they provide a controlled comparison in which incorporation history is varied while dopant identity is fixed. The broader literature contains many additional preparative routes for ceria-based materials, but direct side-by-side comparisons that isolate the pathway variable in this way remain relatively scarce.

This matters because oxygen vacancies in CeO_2_ are not created in an abstract chemical vacuum. Their final concentration, accessibility, and distribution depend on how the fluorite lattice accommodates the incoming aliovalent cation during crystal growth, post-synthetic deposition, and any subsequent reorganization. Hydrothermal incorporation favors dopant participation during nucleation and lattice formation; impregnation introduces the dopant only after the host has already formed; and the combined route allows the dopant to participate in both stages. These differences control how substitutional incorporation, local lattice distortion, and defect-state reorganization are realized in the final material [15,31,32,33,34,35]. For this reason, route should be treated as an active design variable rather than as a background synthesis detail.

The first consequence of route dependence appears at the structural level. Across hydrothermal-, impregnation-, and combined-route Y-doped CeO_2_ series, all samples retain the cubic fluorite phase, indicating that the host framework remains crystallographically comparable within the reported route-controlled comparison despite the different incorporation pathways [15]. At the same time, the diffraction peaks shift toward lower angles after Y introduction, and the extent of the shift varies with route. This means that all three pathways produce Y-containing fluorite solid solutions, but they do not produce equivalent lattice responses. Hydrothermal incorporation generates the largest lattice expansion, impregnation alone the smallest, and the combined route an intermediate response. Already at the crystallographic level, this shows that route changes both the extent and likely the mode of dopant incorporation within the CeO_2_ lattice. The problem is therefore not simply whether Y is present, but how Y becomes part of the defect-bearing host.

XPS analysis reinforces this interpretation. In the source study, wide-scan survey spectra were used to confirm Y incorporation in all three route-dependent samples, and the Y 3d signal was assigned to trivalent Y ions [15]. More importantly, the corresponding high-resolution Ce 3d and O 1s regions were interpreted to track mixed cerium valence and oxygen-species evolution, respectively. The Ce 3d analysis indicates that the relative Ce^3+^ concentration varies across the three pathways rather than remaining fixed at a given nominal dopant identity, while the O 1s analysis shows the same route-dependent ordering in oxygen-defect concentration. Here, the XPS data are used at the level of defect-state interpretation rather than reproduced as additional raw spectral panels. The assignments followed in the source study are consistent with widely used XPS analyses of ceria-based oxides, where Ce 3d deconvolution is employed to evidence mixed Ce^4+^/Ce^3+^ states and O 1s fitting is used to distinguish lattice oxygen from vacancy- or adsorbate-related oxygen species, following standard charge referencing and component-assignment practice reported in the XPS literature [36,37,38]. These results show that route affects not just how much Y is nominally present, but how strongly the Ce^4+^/Ce^3+^ balance and oxygen-defect population are shifted. In other words, the final defect state is not uniquely defined by the formula “Y-doped CeO_2_”; it is route-specific. This conclusion fits well with broader process-sensitive studies on doped ceria and related oxides, where preparation history is known to influence lattice accommodation, local heterogeneity, and defect distribution even at comparable overall compositions [31,32,33,34,35].

Raman spectroscopy gives an even more direct picture of this route dependence. In all cases, the fluorite F2g mode near 464 cm^−1^ is retained, confirming that the host structure remains cubic CeO_2_ [15]. However, the defect-induced Raman band near 592 cm^−1^, associated with substoichiometric CeO_2−x_ units and oxygen-vacancy defects, changes markedly with the doping pathway. The relative vacancy concentration, estimated through the I592/I464 ratio, where I592 is the intensity of the vacancy-related Raman band near 592 cm^−1^ and I464 is the intensity of the fluorite F2g mode near 464 cm^−1^, rises most strongly under hydrothermal incorporation, least strongly under impregnation alone, and to an intermediate level under the combined route [15]. This sequence shows that pure CeO_2_ already contains intrinsic vacancy defects, that Y doping generates additional extrinsic vacancies in all three cases, and, most importantly, that the extent of vacancy creation depends strongly on route. Raman therefore shows clearly that route is a genuine structural variable in defect engineering rather than a procedural footnote.

**Figure 5:**
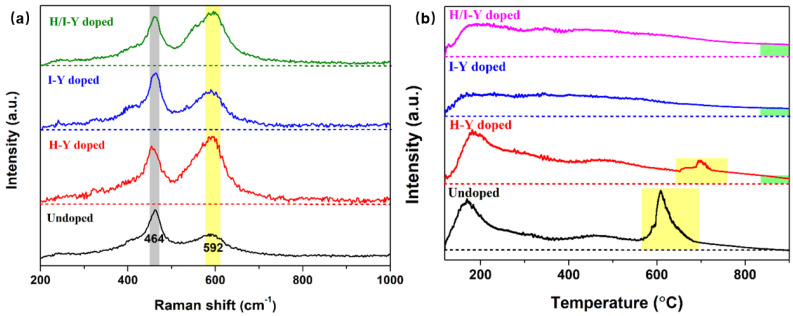
Route-dependent defect-state formation and oxygen-storage behavior in Y-doped CeO_2_. (**a**) Raman spectra of undoped CeO_2_ and Y-doped CeO_2_ prepared by hydrothermal, impregnation, and combined hydrothermal/impregnation routes, showing that the fluorite F_2_g mode is retained while the vacancy-related band near 592 cm^−1^ varies with the incorporation pathway. (**b**) O_2_-TPD profiles of the corresponding samples, revealing route-dependent oxygen-release behavior from surface/subsurface oxygen and bulk lattice oxygen. Comparison of OSC values for the same series, showing that the oxygen-storage response follows the sequence hydrothermally doped CeO_2_ > combined-route doped CeO_2_ > impregnation-doped CeO_2_ > undoped CeO_2_. Together, these panels demonstrate that the final defect state of Y-doped CeO_2_ is controlled not only by dopant identity but also by the incorporation pathway, which determines vacancy-related Raman signatures, oxygen-release behavior, and the resulting OSC. Combined from Ref. [15].

As shown in Figure 5b, the oxygen-release behavior measured by oxygen temperature-programmed desorption (O_2_-TPD) confirms that these spectroscopic differences are not merely formal descriptors but have direct consequences for oxygen handling. For undoped CeO_2_, the O_2_-TPD profile contains low-temperature desorption near ~170 °C and a broader high-temperature desorption region centered around ~600 °C, corresponding broadly to surface/subsurface oxygen and bulk lattice oxygen, respectively [15]. After Y doping, the overall amount of desorbed oxygen increases in all three cases, but the profile shapes also differ. For the impregnation and combined routes, the asymmetrical high-temperature peak becomes smoother, indicating that oxygen desorption is shifted more strongly toward surface and subsurface regions. Meanwhile, all three Y-doped profiles remain above the baseline at higher temperature, suggesting sustained oxygen desorption from the interior of the CeO_2_ lattice and thus the presence of extrinsic oxygen vacancies created by Y substitution [15]. These observations are consistent with the structural and spectroscopic evidence: route-dependent incorporation changes not only vacancy concentration, but also the manner in which oxygen is stored and released across the lattice.

This route dependence becomes fully explicit once the OSC values are quantified. As shown in Figure 5, route-dependent Y incorporation leads simultaneously to different vacancy-related Raman signatures and different oxygen-release behaviors, and these differences are directly reflected in the final OSC values [15]. The reported OSC sequence is hydrothermally doped CeO_2_ > combined-route doped CeO_2_ > impregnation-doped CeO_2_ > undoped CeO_2_. Relative to undoped CeO_2_, the improvements are substantial, but more importantly, the ordering follows the same route-dependent trend seen in the Raman vacancy results. This agreement links route, defect signature, and oxygen-storage response within a single consistent framework. The same Y dopant, introduced through different pathways, gives different lattice expansion, different Ce^3+^ fractions, different vacancy concentrations, different oxygen-release behavior, and ultimately different OSC values. This ordering is mechanistically consistent rather than merely empirical. Hydrothermal incorporation allows Y to participate during nucleation and lattice growth, which favors deeper substitutional incorporation, a stronger lattice response, and more effective defect-state reorganization. Impregnation, by contrast, introduces Y after the CeO_2_ framework has already formed, so the perturbation of the host lattice is weaker and the resulting vacancy-related response is correspondingly smaller. The combined route remains intermediate because the second incorporation step does not simply add to the first one; instead, it partially redistributes a defect state that has already been established, producing a final ensemble that is more defect-active than impregnation alone but less coherently accommodated than the hydrothermal case. This intermediate behavior is plausibly associated with a less uniform final Y distribution and a higher probability of surface-biased or partially associated defects after the second incorporation step, so that total Y introduction does not translate directly into the largest population of reversibly accessible vacancies.

Taken together, the route-dependent Y-doping case changes the way doped CeO_2_ is interpreted. In the single-doping section, the central question was how aliovalent substitution alters lattice and defect chemistry. In the co-doping section, the question became whether simultaneous perturbation of both sublattices could amplify that effect. Here, a different issue emerges, as follows: even when chemistry is held nominally constant, the final defect state still depends on process history. It means that dopant selection alone is not an adequate design principle for oxygen-storage CeO_2_. A nominally identical composition may correspond to different defect ensembles depending on whether the dopant is incorporated during crystal growth, deposited afterward, or introduced in both stages.

This also shows why a purely composition-centered view of oxygen storage is incomplete. If the same dopant can produce different OSC values simply because the incorporation pathway changes, then vacancy concentration itself must be understood as a route-conditioned quantity rather than as an intrinsic material constant detached from synthesis history. The hydrothermal route appears to favor the strongest coupling among substitutional incorporation, lattice expansion, vacancy generation, and oxygen mobility, whereas impregnation alone, although still beneficial relative to undoped CeO_2_, produces a weaker reorganization of the defect state [15]. The combined route further shows that adding an additional incorporation step does not necessarily exceed the most effective primary pathway, which means that route effects are not additive in any simple sense. They reflect how the CeO_2_ framework forms, reorganizes, and accommodates aliovalent substitution over the course of synthesis. The route-dependent case therefore leads directly to the central argument developed here, as follows: oxygen storage in doped CeO_2_ cannot be interpreted satisfactorily through a single variable, but instead emerges from a coupled descriptor set involving lattice response, redox-active cerium species, oxygen-vacancy abundance, and dopant incorporation pathway.

## 5. What Really Governs OSC? From Single Descriptors to Coupled Descriptors

These three cases converge on the same conclusion: oxygen storage in doped CeO_2_ cannot be predicted from dopant identity or nominal vacancy count alone. Rare-earth single doping shows that even within a common trivalent series, the useful OSC window is bounded by how the fluorite lattice accommodates size mismatch and redistributes Ce^3+^/Ce^4+^ alongside vacancy generation. Cation–anion co-doping then demonstrates that simultaneous perturbation of both sublattices can amplify this response, but only within a narrow region where additional defect creation remains structurally and electronically compatible with reversible redox cycling. Finally, route-dependent Y incorporation shows that identical nominal composition does not guarantee identical defect structure, because synthesis history determines where the dopant enters, how vacancies are distributed, and how much of the resulting defect population remains reversibly accessible [13,14,15].

These comparisons are used to extract a descriptor logic that is consistent with broader defect-chemistry analyses of ceria and related fluorite oxides [5,7,12]. In this wider literature, oxygen-vacancy concentration, Ce-centered electron localization, dopant–vacancy association, local structural relaxation, and oxygen-exchange accessibility are treated as coupled quantities rather than isolated variables. The behavior discussed here is not unique to the three focal datasets; rather, these cases make the broader oxygen-storage logic easier to see. This interpretation is also consistent with independent studies on doped and mixed-oxide ceria systems, which likewise report finite OSC optima, nonmonotonic relations between vacancy-related signals and functional response, and strong coupling among lattice relaxation, redox redistribution, and oxygen exchange [1,5,8,17,18,19,20,21].

Seen this way, the proposed descriptor set is best treated as semi-quantitative and hierarchical rather than as a predictive formula. At the first level, lattice accommodation and vacancy accessibility define whether useful defect construction remains possible within the fluorite host. At the second level, Ce^3+^/Ce^4+^ redistribution and dopant incorporation pathway determine how strongly the oxygen-active defect population can participate in reversible redox exchange. This framework does not replace more rigorous predictive models, but clarifies which variables should be considered together when interpreting or comparing OSC behavior across doped-ceria systems. The practical question is therefore not how to combine ionic radius, vacancy concentration, or pathway history into a single equation, but how to identify the regime in which these descriptors remain mutually compatible.

At a deeper level, these coupled descriptors are governed by electronic as well as structural factors. Vacancy formation, electron localization on Ce, defect association, and even incipient dopant segregation are constrained by the electronic chemical potential of the oxide, which is why the concept of Fermi-level engineering is relevant in this context [7,39]. Accordingly, oxygen-vacancy formation should be regarded as a dominant but not exclusive compensation channel in doped CeO_2_, particularly at higher dopant loadings where simple isolated-defect pictures lose rigor and alternative responses such as vacancy clustering, stronger dopant–vacancy binding, incomplete incorporation, or partial segregation become increasingly competitive [5,12]. Here, these non-ideal processes are treated as mechanistic boundary conditions rather than as effects directly quantified in all three model systems. In particular, the current representative datasets do not directly quantify the formation of neutral dopant–vacancy pairs, trimers, or related clustered complexes at higher loading. These are therefore discussed here as plausible defect-chemical limits rather than as experimentally resolved species within the present three case platforms. This boundary matters because it marks where a purely vacancy-centered interpretation may begin to fail.

## 6. Conclusions and Outlook

The main argument developed here is that OSC in ceria is better interpreted through a coupled descriptor framework than through separate discussions of rare-earth doping, cation–anion co-doping, and route-controlled incorporation. In this framework, lattice accommodation sets the structural boundary for defect introduction, redox redistribution determines how much of the cerium sublattice remains electronically responsive, oxygen-vacancy abundance must be distinguished from the reversibly accessible fraction of vacancies s reversibly accessible, and incorporation pathway determines the final spatial and thermodynamic state of those defects. This is why finite OSC optima are common, as follows: beyond a certain perturbation level, additional dopant input no longer increases useful oxygen exchange, but instead promotes stronger defect association, over-distortion, or less reversible defect states.

At the same time, the discussion here does not imply that all aspects of stability or long-term functionality are already resolved for the representative systems discussed here. Where morphology appears largely retained within a given composition-controlled or route-controlled series, such statements should be understood in the limited structural context of the reported datasets rather than as a universal guarantee of long-term stability. In particular, the evolution of OSC over many tens or hundreds of redox cycles remains an essential dimension for future validation, because defect accessibility, redox reversibility, and structural accommodation may diverge progressively under repeated operation even when the initial descriptors appear favorable.

Future work should therefore move beyond composition-based screening alone. Operando spectroscopy, quantitative defect thermodynamics, and synthesis-resolved structural analysis are needed to distinguish total defect content from the fraction of defects that actually participates in reversible oxygen storage. Equally important will be explicit tracking of dopant clustering, vacancy association, and possible partial segregation or exsolution at higher loadings, because these processes can reduce OSC even when nominal dopant concentration continues to rise. In parallel, defect design in ceria would benefit from closer integration of electronic-structure thinking, including Fermi-level control and competing compensation pathways, so that vacancy creation, redox response, and incorporation history are optimized as a coordinated system rather than adjusted one variable at a time.

The focus on CeO_2_ should also be understood in this light. Ceria provides one of the clearest fluorite model systems in which oxygen storage can be discussed through the coupled evolution of lattice accommodation, redox redistribution, and defect accessibility. Other oxygen-storage oxides, including perovskite-type systems, are also highly relevant and may ultimately offer useful comparative contrasts in terms of structural flexibility, oxygen transport, and redox chemistry. Such cross-family comparison is scientifically worthwhile, but it lies beyond the present scope and is best developed as a separate perspective once a similarly controlled descriptor framework has been established across those oxide classes.

## Data Availability

No new data were created or analyzed in this study. Data sharing is not applicable.
